# Methadone treatment providers’ views of drug court policy and practice: a case study of New York State

**DOI:** 10.1186/1477-7517-10-35

**Published:** 2013-12-05

**Authors:** Joanne Csete, Holly Catania

**Affiliations:** 1 Open Society Foundation, 21-24 Millbank, SW1P 4QP London, UK

**Keywords:** Drug courts, Methadone, Buprenorphine, Criminal law, Incarceration

## Abstract

**Background:**

Specialized drug treatment courts are a central part of drug-related policy and programs in the United States and increasingly outside the U.S. While in theory they offer treatment as a humane and pragmatic alternative to arrest and incarceration for certain categories of drug offenses, they may exclude some forms of treatment–notably methadone maintenance treatment (MMT). We sought to understand from the perspective of treatment providers whether this exclusion existed and was of public health importance in New York State as a case example of a state heavily committed to drug courts and with varying court-level policies on MMT. Drug courts have been extensively evaluated but not with respect to exclusion of MMT and not from the perspective of treatment providers.

**Methods:**

Qualitative structured interviews of 15 providers of MMT and 4 NGO advocates in counties with diverse court policies on MMT, with content analysis.

**Results:**

Courts in some counties require MMT patients to “taper off” methadone in an arbitrary period or require that methadone be a “bridge to abstinence”. Treatment providers repeatedly noted that methadone treatment is stigmatized and poorly understood by some drug court personnel. Some MMT providers feared court practices were fueling non-medical use of prescription opiates.

**Conclusions:**

Drug court practices in some jurisdictions are a barrier to access to MMT and may constitute discrimination against persons in need of MMT. These practices should be changed, and drug courts should give high priority to ensuring that treatment decisions are made by or in close consultation with qualified health professionals.

## Background

Addiction to opiates is a significant public health problem in the United States. An estimated 620,000 persons used heroin at some time in the previous year in 2011 compared, for example, to 373,000 in 2007 [1]. The number of persons estimated to be dependent on pain relievers–mainly opiates–increased from 1.5 to 1.8 million from 2002 to 2011, representing the second most prevalent drug dependence after marijuana, according to the U.S. government’s assessment [1].

Decades of research and practice have established that opioid dependence can be treated successfully and cost-effectively. Maintenance (continuous) therapy using methadone, an opioid agonist, has been used in the U.S. since the 1960s [2]. Methadone maintenance therapy (MMT) is recognized by the U.S. Institute of Medicine as well as by the World Health Organization (WHO) to reduce cravings for illicit opioids, reduce crime linked to drug use, reduce deaths from overdose, reduce HIV risk behaviors, and help patients stabilize their lives and sustain productive activity [3,4]. Since 2002, buprenorphine has also been authorized for treatment of opioid dependence in the U.S. and is in wide use [5]. Methadone and buprenorphine are included for their therapeutic use on the WHO Model List of Essential Medicines [6].

In spite of the strong scientific evidence base for it, as of 2008 only an estimated 10 percent of those who could benefit from it had access to MMT [7]. This access is hampered by many factors, including the failure of public and private insurers to cover this treatment [8,9]; the requirement that methadone ingestion be directly observed in a specialized facility every day [9]; the lack of providers or nearby providers [9,10]; the lack of availability of opioid dependence therapies in U.S. prisons and pretrial detention facilities [11,12]; and resistance of local authorities and citizens to having methadone clinics in their neighborhoods [13].

Another factor is looming large as a determinant of access to opioid dependence treatment in the U.S.–the rapid proliferation of drug courts. As of June 2013 there were 2734 drug treatment courts in the 50 states and U.S. territories [14], up from only one in 1989 and 665 in 2000 [15]. The National Association of Drug Court Professionals, which is part of the federally funded National Drug Court Institute, promotes the establishment of drug courts and other “problem-solving” courts across the U.S. [16]. U.S. officials also promote the drug court model heavily outside the U.S. [17], as also observed by one of the authors, JC, who was present as U.S. drug control policy officials participated in the National Association of Drug Court Professionals and UN Office on Drugs and Crime’s side event, “Alternatives to incarceration: providing health-based services for drug-dependents in the criminal justice system” at the annual session of the UN Commission on Narcotic Drugs, 13 March 2013. At least 15 countries have drug courts along the lines of the U.S. model [18].

While drug courts’ policies and practices are not uniform even within the same state, they generally share the objective of diverting non-violent drug users from prison into treatment for drug dependence [19]. Most drug courts require patients in court-mandated treatment to be followed closely by the court, including frequent urinalysis for drug testing and “non-adversarial” appearances before a judge.

U.S. drug courts have been widely evaluated with respect to rates of recidivism and “graduation” from treatment and recidivism [20,21], but not with regard to patterns of or access to treatment for opioid dependence. While drug courts in theory could facilitate access to opioid maintenance therapy, the available survey data indicate this is not the case. Of U.S. counties surveyed in 2001, 20 percent said that their drug courts prohibit any kind of medically assisted treatment including MMT, 61 percent said medically assisted approaches were limited or were offered only for a period specified by the court, and 19 percent had no policy [22]. A more recent survey to which 103 drug courts responded indicated that 50 percent allowed no MMT or other opiate agonist medication under any circumstances, and 34 percent allowed MMT in some circumstances, including when defendants were already methadone patients [23].

In California in 2000, a drug court judge who believed that methadone fails to “break the cycle of addiction” required a defendant to stop MMT against the man’s wishes and against the advice of his treating physician. The defendant died two months later of a heroin overdose [24]. Information on such cases is difficult to collect since persons needing methadone who may be denied access to the courts are often lost to follow-up.

The purpose of this paper is to investigate in another U.S. state, New York, the practice of drug courts with respect to MMT and the impact of those practices on access to MMT. Few evaluations of drug courts have included the perspective of providers of treatment services, as this study has done. We sought to investigate treatment providers’ understanding of drug courts’ MMT policies and whether MMT providers were regularly consulted by drug court personnel in the disposition of cases of defendants with opioid dependence.

## Methods and setting

The State of New York was chosen for this work because it has the second largest drug court presence in the country after California, with 148 drug courts of various kinds in operation as of May 1, 2013 [25]. As of 2010, all 62 New York counties had a drug treatment court except the county that contains only state forest land [26]. In 2009, the number of participants in drug court programs averaged 7253 per month [26]. Of these, 22 percent had opiates as their drug of choice [26].

We intended to interview MMT providers in jurisdictions with varying drug court policies with respect to MMT and to include the largest urban areas in the state, but we were unable to visit all counties with MMT programs and so reviewed information available on MMT practices. Few courts stated their policies in publicly available documents, and we found it was not easy to get this information from court coordinators on the telephone. The only readily available information on MMT policies of drug courts in New York was from a 2003 survey, indicating that three of five counties in New York City (Manhattan, Brooklyn and Queens) excluded MMT unless patients withdrew from methadone in a period fixed by the court; courts in Erie County (including the city of Buffalo) stated that they followed the recommendations of treatment providers; and courts in Suffolk County (Long Island), Onondaga County (home of Syracuse) and Bronx County said they did not require methadone abstinence for participation [27]. Among the few courts with public statements of their policies were the Manhattan and Brooklyn drug courts, which stated their MMT policies in participant handbooks.

The authors interviewed 15 treatment providers in the following New York counties, which represented a range of stated policies on MMT: Brooklyn (Kings County), Manhattan (New York County), Bronx, the counties of the judicial Western District (Erie, Allegany, Wyoming, Genesee, Niagara, Orleans, Chattauqua, and Cattaraugus), Onondaga, Nassau (Long Island), and Albany. One of the persons interviewed was also in the process of starting a new methadone service in upstate Jefferson County. Treatment providers were identified through the registry kept by the federal Substance Abuse and Mental Health Services Administration (SAMHSA). Of the treatment providers interviewed, six were directors of clinics, and the others were senior clinic staff members. In counties outside New York City, methadone clinics are few and are well known. Some of the clinics also provided buprenorphine, but the majority clientele for all facilities included in the study were methadone patients. As buprenorphine is mostly prescribed by individual practitioners and not through special stand-alone facilities, we did not attempt to sample the many providers of buprenorphine maintenance therapy in the state.

For clarification of drug court policies, two drug court coordinators–full-time staff members of drug treatment courts–and one sitting drug court judge were also interviewed. We also spoke with representatives of four non-governmental organizations that have studied drug court practices from a human rights or legal service perspective and have testified on drug courts at public hearings in the state legislature.

The questionnaire used for treatment providers is shown in the Appendix. Questionnaires for NGO staff and drug court personnel were similar. Questions were asked in an open-ended way. Interview notes were transcribed and content analysis using NVivo was planned, but in general the longer narratives that we anticipated did not materialize as in some jurisdictions, there was not extensive interaction between the drug courts and treatment providers. We analyzed transcripts with respect to the key questions of whether MMT was permitted as a court-mandated therapy, whether a mandatory tapering-off period was imposed, and the degree to which treatment providers or other specialized health professionals were involved in drug court decisions about mandated treatments. The few other themes that emerged are noted in the results.

It was the intention of the authors to interview methadone patients with drug court experience, whom we planned to identify with the collaboration of treatment providers and seek their informed consent to be interviewed. In the end, most of the stories of patients we heard were of persons excluded from drug courts, and the persons in question could not be found because they were unable to continue in supervised treatment. As a result, we spoke to only two patients, one in Brooklyn and one in Nassau County, and the director of a national patient advocacy group based in New York.

Buprenorphine maintenance is also offered by some methadone maintenance providers. However, buprenorphine treatment, unlike methadone, can be provided for opioid dependence maintenance therapy in a physician’s office rather than in specialized facilities where patients have to present themselves every day. As a result, most MMT providers are dealing mostly with methadone. MMT providers are readily identifiable, unlike the many private physicians who provide buprenorphine. For this reason, buprenorphine is not a principal focus of this study.

The research protocol and questionnaires were approved by the Institutional Review Board of the Columbia University Medical Center where one author (JC) was a faculty member at the time of the research.

## Results

### Policies and practices of the courts

In our sample, only one court (Bronx County) had a clear practice of welcoming persons already receiving MMT and including MMT as an option for court-referred treatment. Albany County was characterized by treatment providers as having zero tolerance for methadone; all MMT patients and persons who wanted to receive methadone or buprenorphine as court-mandated therapy were excluded.

The Manhattan and Brooklyn drug courts, among the largest in the state, required any existing methadone patients before the court to withdraw from MMT. The two policies, according to their respective handbooks, are the same [28,29]. To participate in the drug court, methadone patients must agree to participate in a phased withdrawal from MMT, of which the first phase includes demonstrating “abstinence from all other substances”, receiving a methadone dose reduced by half, and having four months “sanction-less time”. The second phase is complete “detox from methadone” as well as abstinence from other substances and unspecified “accumulated drug-free time” of which the duration depends on the offense with which the person is charged.

The Western District, including eight counties and the city of Buffalo, which reportedly followed the guidance of treatment providers, was typical of those districts with a general policy of allowing MMT only for a fixed period as a “bridge to abstinence”. The Western District was of particular interest because a leading drug court judge in the district, the Hon. Robert Russell, had been board chairman of NADCP, which has a resolution on the benefits of MMT not as a bridge to abstinence but for whatever period is needed [30]. Asked about the difference between the 8^th^ District’s policy and the NADCP policy, Judge Russell asserted that “expressing the aspiration that all people will be drug-free” is not counter to the spirit of the NADCP policy.

In the Bronx, where the drug court welcomed methadone patients, treatment providers noted, however, that the position of the drug court was undermined by the policy and practices of another state-funded program operating in the criminal courts, Treatment Accountability for Safer Communities (TASC)^a^. TASC is meant “to integrate alcohol and substance abuse treatment into justice processing to provide continuous treatment and supervision for substance-involved justice populations, and there is a large TASC-supported program in the Bronx [31]. According to treatment providers, persons receiving services from TASC-supported community organizations are prohibited from MMT. Treatment providers elsewhere in New York City also reported that TASC programs’ intolerance for MMT was a problem for their patients.

It was predicted by state lawmakers that the reform in 2009 of New York’s strict drug laws -- known as the Rockefeller drug laws–would lead to an expansion in demand for participation in drug treatment court programs [32]. Treatment providers with experience before and after the reforms were asked whether they perceived that the reforms were associated with a greater opportunity for opioid-dependent defendants in criminal and drug courts to be diverted to community-based MMT. None reported evidence of any increase in referrals to MMT by the courts.

### Obligatory rapid tapering as a medical concern

MMT providers in most counties expressed concern about the widespread idea in drug courts that MMT is meant only as a bridge to abstinence–i.e. that treatment cannot be maintained over a long period or indefinitely. Treatment providers were especially concerned about court orders for abrupt “tapering” to abstinence for persons already in MMT. Some treatment providers, including in Manhattan and Brooklyn, reported that they and their staff spent a great deal of time fighting these orders. Case by case, they mobilize doctors’ statements and advocate with court officials. For some, this is a major burden on their staff that has grown with the prominence of the drug courts. Also of concern was the courts’ usurping the role of the treating physicians in deciding when patients should stop taking their medication as prescribed.

Some providers said that they manage to win these arguments at least some of the time, and the fight, though time-consuming, is worth it to give patients access to care they need. Others indicated that they know they can’t win and have largely given up. A treatment provider in Albany County said that his clinic even had the support of the Albany City Police in advocating for MMT, but the court would not entertain this possibility. Treatment providers asked to explain why they thought the drug courts did not tolerate extended MMT attributed these attitudes to the personal biases of judges and drug court coordinators. “Methadone always has this stigma associated with it”, said one provider. “People can’t think of it as medicine”.

We heard from some treatment providers that MMT patients and potential patients who wanted to be in the drug court or in a TASC program were finding alternative ways to get the opioids they needed. One provider told us: “People are finding ways to get around the courts’ methadone prohibition by getting prescriptions for short-acting analgesics like oxycodone. The judges allow those prescriptions, but not methadone or buprenorphine. We’re seeing patients come back to the methadone clinic after going through these court-mandated programs for help with getting off the prescription drugs. Addiction to prescription drugs is one of our biggest problems”.

Upon referral from a treatment provider, we interviewed a woman aged 33 years who had come to drug court after being arrested for felony drug possession. Like all other defendants in New York, to be eligible to participate in drug court she was required to enter a guilty plea to the original criminal charge and in her case faced a mandatory 5-7-year prison sentence if she failed to complete the court’s requirements. She had a history of opioid dependence but found, while in drug court, that buprenorphine maintenance therapy was effective for her and enabled her to keep a job. Nonetheless she was required by the court to be “clean”–that is to stop taking buprenorphine -- within 45 days before she could “graduate” from drug court. She was supervised by four different judges who cycled through the court over 18 months, and completed a six-month stay in a residential treatment facility, followed by a year of frequent urine toxicology tests on demand. She told us: “I had to leave my job every time they called me in for a tox test. They would call me at 9:00 or 9:15 in the morning and demand that I be at the court by 4:00 or 5:00 that day”. She had a supportive employer and social network and managed through remarkable personal initiative to stop buprenorphine therapy abruptly for the required period so that she could graduate from drug court. She said: “I want to take the medication more than I want to get high. I don’t want to live like that anymore and I will go back into treatment as soon as my case is over”.

### Waiting lists

Treatment providers in upstate counties reported that there were waiting lists for places in MMT programs, averaging several months. The number of MMT programs and places within programs is restricted by state and local regulations and affected by stigma and community resistance, resulting in demand far outweighing availability. In Onondaga County, treatment providers could sometimes reserve a few spots for people referred by the drug courts, but this was not always possible and not at all possible in other upstate counties. As treatment providers noted, even if the courts were friendlier to MMT, waiting lists would be an impediment unless there were assistance from the state enabling clinics to expand or to retain slots for court-referred patients. One director said to us: “I have 100-150 people waiting to get into treatment on any given day. How can I bump someone who’s committed a crime ahead of them?” Existing MMT patients who find themselves in the drug court would presumably not face this barrier, though in some jurisdictions they would face arbitrary “tapering off” deadlines.

### Communication between courts and treatment providers

Treatment providers reported a variety of models of regular communication or contact between themselves and drug court personnel. In the Western District, treatment providers may choose to station staff members at the courthouse on the days the drug treatment court is in session. Health or social service staff who are present are invited to help evaluate the treatment possibilities for drug court participants. Treatment providers in Onondaga County said they had regular meetings with drug court personnel. In other counties, MMT providers reported that they did not generally have contact with drug court staff unless they needed to go to court to argue on behalf of patients. In one of the larger counties, a provider who has worked at the same MMTP since 1970 told us: “It used to be if a judge ordered a person into MMT, we’d go back to court and fight that. Now we’re left out. We need a single system where MMT is a part of the treatment options. With drug courts, the choice is taper or jail. The science doesn’t count”. Another said: “It varies from jurisdiction to jurisdiction. We see typically 5-10 people a year forced by the courts to reduce their [methadone] dose to zero”. A provider in a smaller county recounted two recent cases of patients being forced to stop taking their methadone by the court: “A patient in her mid 20’s, doing well in MMT was ordered by the judge to stop taking her methadone. She was facing a lot of jail time and somehow managed to do it long enough to graduate the drug court program. She went back into MMT when she got out. Another patient in a different county was ordered to stop MMT and we don’t know what happened to him. We lost contact”. He added: “We don’t have the staff to fight those battles in court like the bigger MMTPs in the City”.

A few said that they invited drug court personnel to visit their facilities but the invitations were not accepted. One provider who has worked in MMT for over forty years said: ‘A few years ago we invited drug court staff to visit our program. They sent their social service people to present to us what they do, but it’s the judges and prosecutors who won’t allow MMT in their program”. Another provider told us: “We do trainings for the courts at the judges’ request. There’s no hierarchical structure controlling the judges and they have very little knowledge of MMT”. The Western District was exceptional in that when the drug court was established, the judge convened a meeting with treatment providers, including the methadone clinic staff in the jurisdiction.

One legal advocate we interviewed had this to say about the relationships between providers and the drug courts: “Treatment providers should be considered the fourth leg of the stool…The drug courts have no rules; the judges make their own rules. The treatment providers need to better understand the criminal justice system and that criminal defense lawyers are natural allies. There’s undue deference to prosecutors and judges and so few defense lawyers understand the literature, science and research that supports their arguments”.

### Methadone for detainees

Methadone treatment is generally not available to persons in jails and prisons in New York, except in Rikers Island, New York City’s main jail. Some of the upstate treatment providers reported that they are sometimes able to get permission from the drug courts for methadone to be brought to existing patients who are in pretrial detention while they are under orders to “taper” and eventually cease methadone treatment.

## Discussion

The research newsletter of the National Association of Drug Court Professionals (NADCP) boasts: “From their inception, Drug Courts embraced science like no other criminal justice program. They endorsed best practices and evidence-based practices…” [21]. The MMT policies and practices of drug courts in several New York counties embody just the opposite: a rejection of science and scientifically sound best practices and raise serious questions about medical ethics. Court-ordered forced withdrawal from MMT and allowing methadone and buprenorphine therapy only as a “bridge to abstinence” are contrary to agreed national and international best practices and are a disservice to people who may benefit from the courts but who also benefit clinically from MMT. As noted by the World Health Organization, a strong body of research supports the conclusion that arbitrary limitation of the period of MMT is clinically counter-indicated [4]. The experience of treatment providers in the state indicates that these policies undermine the ability of the courts to serve many of the very persons they were designed to serve.

The practices documented here run contrary to those of bodies with the mandate of guiding and supporting drug courts in the US. In 2011, NADCP, a professional association supported heavily by federal agencies, published a resolution noting that drug courts should not “impose blanket prohibitions against the use of [MMT] for their participants” and that court decisions in this regard should be based on “particularized assessment in each case” [30]. An earlier position paper by the National Drug Court Institute, the “professional services” arm of NADCP, adopted a position statement that methadone patients “should not be required to withdraw from a medication that improves their lives” any more than people who need them should be forced to withdraw from treatment for cardiovascular conditions (This statement no longer appears on the NDCI web site but is reproduced by Parrino in 2003) [33]. Allowing methadone therapy only for arbitrarily specified periods defies the accepted evidence that continuity of MMT over a long period and even indefinitely is well indicated for some patients [4]. A more recent “best practices” document of NADCP notes that in view of “numerous controlled studies” showing “significantly better outcomes” associated with methadone and buprenorphine therapy, “a valid prescription for such medications should not serve as the basis for a blanket exclusion from the Drug Court” [34].

In 1997, the National Institutes of Health convened a consensus panel that strongly recommended greater access to MMT for people who need it. The panel stressed that “[a]ll opiate-dependent persons under legal supervision should have access to methadone maintenance therapy, and the U.S. Office of National Drug Control Policy and the U.S. Department of Justice should take the necessary steps to implement this recommendation” [35]. This recommendation has never been implemented on a national or local level and is plainly not being followed by some drug courts. Practices of the courts also fly in the face of the recommendations of United Nations expert bodies on health and drugs as well as highly regarded expert reviews of the Cochrane Collaboration that have investigated both methadone and buprenorphine maintenance with respect to effectiveness and safety [4,36,37]. Numerous studies have shown that MMT keeps people with opioid dependence in treatment longer than the abstinence-based therapies apparently favored by some drug courts, and dropping out of treatment is a major risk factor for overdose [38,39].

The State of New York has addressed MMT in guidelines on recommended drug court practices, as follows:

Methadone maintenance therapy can be a controversial topic when utilized in the criminal justice context…. Criminal justice professionals tend to view methadone as another drug that is addictive and subject to misuse…. Drug court programs should make their decisions about [MMT] in the same manner that they make other treatment-related decisions, in close consultation with the treatment professionals on their team [40].

The experience of the treatment providers we interviewed indicates that this guideline is being routinely violated in some New York counties as treatment providers are not only not consulted in clinical decision-making of the courts but are sometimes compelled to make special appeals to the courts to respect clinical norms and ethics. Decision-making about treatment for drug dependence should always be made by health professionals with specialized knowledge of treatment efficacy and safety.

The perception of treatment providers in this study that the criminal justice system was biased against MMT was corroborated by findings of an unpublished 2011 analysis by the New York State Office of Alcoholism and Substance Abuse Services (OASAS), which found that while “40% of admissions to all drug treatment services were referrals from criminal justice agencies, only 5% were in opioid treatment programs (OTPs)”. This data was presented by OASAS director Arlene Gonzales-Sanchez at a meeting of the Committee of Methadone Program Administrators of New York State on May 15, 2011 in New York City in which one of the authors, HC, was present. Treatment providers’ conclusion that methadone and other medication-assisted treatment for opiate dependence are stigmatized in the drug courts also corresponds to the finding of Matusow and colleagues that MMT was sometimes seen as just a “new addiction” to replace an old one [23].

In view of the stigmatization of methadone as a treatment option and of methadone patients, the state has a responsibility to protect access to this treatment as a matter of legal protection of the rights of patients. The Americans with Disabilities Act (ADA) of 1990 has been used to defend the placement of methadone clinics in locations where residents have objected to them [41] and to prohibit discriminatory dismissal of workers who are methadone patients [42]. Similarly, there may be grounds under ADA to assert that the rejection of MMT as a treatment option for drug courts is discriminatory. Apart from legal responsibility, the state should see as costly and problematic the loss of persons with opiate dependence to scientifically sound and supervised care or their diversion to illicit use of prescription opiates. The human cost of denial of this treatment for the individuals kept from medicines that can stabilize their lives and keep them from having to seek drugs in illicit markets is enormous. As the World Health Organization [4] and an important body of research [43] has shown, beyond its direct effect in stabilizing opioid cravings, MMT helps people have stable family lives, maintain employment, and be functioning members of their communities.

D’Aunno asserts that a range of barriers to the use of “empirically established treatment” of opioid dependence in the US includes (1) limited resources; (2) poorly designed policies, organizations structures and incentives; (3) organizational and individual values, beliefs and norms; and (4) individual deficits in training, skills and motivation [44]. All of these factors may be relevant to drug courts as a barrier to access to MMT. If limited resources result in waiting lists for MMT slots, it is that much harder to advocate for inclusion of methadone as an option for court-mandated therapy. Policies that incentivize exclusion of needy patients should be corrected. Individual values of judges and drug court personnel that reinforce practices that deviate from clinical and scientific norms should be addressed. Where these values are a result of inadequate technical capacity, training should be brought to bear and measures should be taken to ensure that qualified treatment providers have a meaningful role in treatment-related decisions of the courts.

Until court practices improve, it would be useful for the state to establish a monitoring system to follow cases of people rejected from drug court participation because MMT is their clinically indicated or preferred treatment, especially to ensure that these persons are able to have access to the care they need. The federally supported National Drug Court Institute or another body should investigate the degree to which drug treatment courts in the US are defying best clinical practice on MMT and the reasons why, so as to inform appropriate policy responses.

This study has several limitations. The inclusion of more counties may have elicited more varied case examples. The experiences of MMT patients in the few drug courts in which they could participate or of those receiving MMT who may have been required by the courts to “taper off” methadone would have been a useful complement to that of treatment providers, but we did not have the means to track down the persons whose stories were referred to by treatment providers. The experiences of persons for whom MMT is clinically recommended but who wish to participate in drug courts that do not allow MMT as a treatment option would be a fruitful topic for further research.

## Conclusion

The MMT practices of New York drug courts do not correspond to the best practice consensus of drug court professional associations, US government health authorities and international authorities. Practices such as those documented here exclude already vulnerable persons from humane and effective treatment to which they are entitled.

The 2009 reform of the Rockefeller-era drug laws in the State of New York included legislative grounding for expanded use of alternatives to incarceration, including court-supervised treatment for drug dependence [26]. Drug treatment courts continue to become more numerous and may handle more cases as the processes associated with this reform are institutionalized. To the degree that drug treatment courts persist in excluding MMT from treatment options, the courts’ effectiveness will be compromised for a key segment of the population they were meant to serve. That this population is already stigmatized and underserved should speak to urgency of the need for federal, state and civil society support for improving the capacity of drug court judges and staff to adopt medically sound practices in an area of drug dependence that benefits from decades of scientific research.

## Endnote

^a^The TASC program was originally established as a federal initiative in drug legislation of 1972 under the name Treatment Alternatives to Street Crime. The State of New York’s Division of Criminal Justice Services now operates task under the name Treatment Accountability for State Alternatives. See the Division’s web site at http://www.criminaljustice.ny.gov/opca/ati_description.htm.

## Appendix: Questionnaire for treatment providers

1. How long have you been working at that site (and situation that date with respect to the start of drug court activities in the jurisdiction)?

2. When the drug court began its activities, did court personnel consult with you or other service providers? If so, what form did that consultation take? Did that consultation include discussion of or information about medication-assisted therapies? If not, were there other ways in which drug court personnel communicated with treatment providers, including on policies related to MMT and buprenorphine?

3. What do you understand to be the policy of the court on MMT and buprenorphine? In your experience, does the policy conform to the practice of the court? If not, in what ways do they differ?

4. Do you or others in your facility have regular contact with court personnel, including prosecutors and defenders? If so, describe the circumstances under which you are likely to be in contact. If you need to be in contact with them, are you able to be? If not, what are the barriers to contact?

5. Have staff members of the court visited your facility? If so, how many times and under what circumstances?

6. Have you spoken to any court personnel about the court’s policies or practices related to medication-assisted therapy? If so, under what circumstances, and what was the result of your interaction?

7. How would you characterize the volume of patients referred to your facility by the courts, and how overall has the drug court’s work changed demand for services at your facility? Have you had to adjust practices or procedures because of the volume or nature of court referrals? If so, how?

8. Have drug court practices and their impact on your facility affected access to or quality of treatment services for patients not mandated by the courts?

9. Have you been required to assist court-ordered patients in withdrawal from MMT or buprenorphine? Can you characterize the results of those procedures in general?

10. How do you see the recent changes in NY State’s drug laws as affecting the drug courts or your services generally? How about with respect to MMT and buprenorphine?

11. [If it isn’t already clear] Generally, how would you characterize the changes that have come about in your work because the drug courts are there? Have they been an overall asset for your services? If so, in what way? If not, why not?

## Competing interests

The authors declare no conflicting or competing interests.

## Authors’ contributions

Both authors contributed to this article and to all stages of the research. Both authors read and approved the final manuscript.

## Authors’ informations

Joanne Csete, PhD is senior program officer for the Global Drug Policy Program. Prior to joining the Open Society Foundations, she was associate professor at Columbia University’s Mailman School of Public Health in New York, where her research focused on health services for marginalized and criminalized populations, especially people who use illicit drugs, sex workers, prisoners and detainees, and people living with HIV.

As the founding director of the HIV and Human Rights Program at Human Rights Watch, Dr. Csete oversaw the building of a body of research on human rights abuses as barriers to access to HIV services and conducted advocacy with many governments on removing those barriers. She was executive director of the Canadian HIV/AIDS Legal Network in Toronto, one of the world’s leading health and human rights research and advocacy organizations. She was previously a senior technical advisor at UNICEF and taught at the University of Wisconsin-Madison. She worked on health programs in Africa for over 10 years.

Holly Catania, JD worked as a public defender in the Manhattan Criminal Courts representing indigent defendants for five years after receiving her law degree from Temple University. Since then, she has spent much of her career advocating for increasing and improving access to opioid addiction treatment and harm reduction to address the health and social harms associated with problematic drug use and punitive drug policies internationally. She has worked with the Drug Policy Alliance and International Harm Reduction Development Program at Open Society Foundations, the International Center for Advancement of Addiction Treatment at Beth Israel Medical Center, and International Doctors for Healthy Drug Policies assisting people in the medical profession, non-governmental organizations and government entities struggling with drug problems and policies to foster humane and evidence-based responses that emphasize health, justice and human rights over prohibitionist policies.

She has co-authored two research papers evaluating the first U.S. state-prison based methadone maintenance treatment program and is the editor of three editions of the About Methadone and Buprenorphine handbook, a patient’s guide and public resource that has been distributed to more than 300,000 people worldwide.
